# Cancer antigen 125 and C-reactive protein inflammatory mediators and uric acid in association with preeclampsia in North Kordofan State, Western Sudan

**DOI:** 10.1371/journal.pone.0280256

**Published:** 2023-01-23

**Authors:** Naglaa Abdelbasit Suliman, Khidir Elamin Awadalla, Khalid Hussein Bakheit, Abdelrahim Osman Mohamed

**Affiliations:** 1 Department of Biochemistry, Faculty of Medicine, University of Kordofan, Elobaied, Sudan; 2 Department of Obstetrics & Gynecology, Faculty of Medicine, University of Kordofan, Elobaied, Sudan; 3 Department of Clinical Biochemistry, Faculty of Medicine, King Abdulaziz University, Jeddah, Kingdom of Saudi Arabia; 4 Department of Biochemistry, Faculty of Medicine, University of Khartoum, Khartoum, Sudan; University of Wisconsin-Madison, UNITED STATES

## Abstract

Pathophysiology of pre-eclampsia depends on a defective trophoblastic invasion of uteroplacental blood vessels that leads to placental ischemia and induction of an inflammatory process within the placenta. This process may trigger the expression of Cancer antigen 125 (CA 125), C-reactive protein (CRP) and uric acid (UA). This research aimed to evaluate the association of serum CA 125, CRP and uric acid with Preeclampsia. The study recruited 200 singleton Sudanese pregnant women, who were divided into three groups: controls (n = 100), mild preeclampsia (n = 46) and severe preeclampsia (n = 54). The study subjects were matched for maternal age, gestational age and body mass index. Blood samples were taken for measurement of the different variables using immune- assay and enzymatic automated chemical analysis. The levels of CA 125 in mild and severe preeclampsia were (21.94±0.749 IU/ml) and (40.78±1.336 IU/ml) respectively, which was significantly different (*P*<0.001) from the control mean (16.48±0.584 IU/ml). There was also a significant difference between the mean levels of CRP in mild and severe preeclampsia (15.17±0.788 mg/L), (31.50±1.709 mg/L) compared with controls (4.79±0.178 mg/L), (*P*<0.01). There was also a significant difference in the mean levels of UA in mild and severe cases (6.44±0.293 and7.37±0.272) in comparison with the controls (4.00±0.061); (*P<0*.001). There were significant differences between severe and mild groups (*P<0*.05). Cancer antigen 125, CRP and UA levels‎ correlated positively with mean arterial blood pressure (MAP) where (r >0.7; *P* < 0.001). ROC curve validates the utility of these biomarkers for monitoring preeclampsia (AUC >0.8; *P* < 0.001). In conclusion CA 125, CRP and UA were significantly higher in preeclampsia compared with the controls. The rise of the analytes was directly associated with the severity of the disease.

## Introduction

Preeclampsia (PE) is defined by the International Society for the Study of Hypertension in Pregnancy (ISSHP) as the presence of new-onset hypertension and proteinuria or other end-organ damage occurring after 20 weeks of gestation [1]. Earlier definition of preeclampsia stated that it is a pregnancy-specific hypertensive disorder with multi-system involvement that originates in the placenta [2]. Preeclampsia remains the main cause of variable maternal and fetal morbidity and mortality in the developing countries [3]. It complicates approximately 5–8% of all pregnancies [4]. It is characterized by the onset of hypertension (BP ≥140/90 mm Hg) and significant proteinuria (300 mg/day) that occurs at or after the 20^th^ week of pregnancy in a previously normotensive woman [5]. One of the proposed theories for the pathophysiology of preeclampsia is the constriction of the spiral arterioles in the placental bed due to the failure of their invasion by trophoblasts. The consequence of this vaso-constriction is placental ischemia causing the release of inflammatory factors in the maternal blood leading to endothelial dysfunction. The clinical picture of the disease results from this endothelial dysfunction [6, 7]. Preeclampsia is associated with increased systemic vascular resistance, enhanced platelet aggregation and activation of the coagulation system [8–12]. Symptomatic pre-eclampsia usually develops after the 20^th^ week of pregnancy with clinical manifestations including hypertension, renal insufficiency in the form of decreased GFR, proteinuria and coagulopathy. In severe forms, patients may develop hemolysis, elevated liver enzymes and low platelet (HELLP syndrome). Seizures, blurring of vision and headaches are complications affecting 5 of every 10,000 live births [12].

The enhanced maternal inflammatory response responsible for endothelial dysfunction may trigger the expression of cancer antigen (CA-125) and C—reactive protein (CRP) as inflammatory mediators. CA-125 is a glycoprotein complex antigen that is expressed by the epithelial ovarian tumor [13, 14]. CA-125 is a marker of peritoneal and pleural diseases [15] and was detected in the serum of many physiological and pathological conditions [16, 17]. The separation of trophoblast from decidua is anticipated as the primary cause of the elevation of CA-125 in the serum of patients with preeclampsia [18, 19]. The role of CA-125 in obstetrics is not fully clarified as most clinical trials recommending its use are generally experimental in nature. There are few clinical studies related to the use of CA-125 in hypertensive disorders of pregnancy with conflicting results [20]. However, some studies reported positive correlations between serum CA-125 concentration and preeclampsia [21–24].

C—reactive protein (CRP) is an acute phase protein produced by hepatocytes in response to the release of pro-inflammatory cytokines. It is a sensitive marker of systemic inflammation [22] responsible for the endothelial dysfunction in preeclampsia [25]. Many studies reported its sensitivity and specificity in the prediction of PE [26–30]. On the other hand, Uric acid (UA) is a major end product of purine catabolism [31]. Hyperuricemia is the most consistent and earliest detectable change in preeclampsia which was reported as a better predictor of fetal risk [32–35]. Hyperuricemia has been related to cardiovascular and renal diseases through the generation of reactive oxygen species (ROS) and subsequent endothelial dysfunction [33, 34, 36–38]. Another study revealed that the level of serum uric acid was not steadily elevated in all women with severe preeclampsia, suggesting that the uric acid was not a useful predictive test for PE [39]. The objective of this study was to assess the levels of CA-125, CRP and UA and to determine their association with preeclampsia in Sudanese pregnant women.

## Materials and methods

This is a case-control study conducted in Elobeid teaching hospital, North Kordofan State, Western Sudan, during the period from December 2017 to December 2020. A hundred patients with preeclampsia attending the antenatal ward and labor room, in the age range (15–50 years old) who fulfilled the criteria for preeclampsia were approached to participate in the study as cases. They were further categorized into mild (n = 46) and severe preeclampsia (n = 54) according to the diastolic blood pressure (<110 or ≥110 mmHg) and (systolic blood pressures <160 or ≥160 mmHg) respectively [40]. A number of 100 normotensive pregnant women, who presented to the same outpatient clinics, were recruited as a control group. Both groups were in the third trimester. Gestational age was calculated from the first day of the last menstrual period and confirmed by early first trimester ultrasound reports in uncertain cases. Blood pressure was measured for all patients and controls with a mercury sphygmomanometer. Patients having persistent high blood pressure ≥140/90 mmHg on 2 or more occasions 6 hours apart with proteinuria ≥ +2 by dipstick or ≥300 mg/day in 24 hours’ urine collection were chosen for this study. Mean arterial blood pressure (MAP) for each subject was determined by the formula MAP = [Diastolic blood pressure + (systolic blood pressure–diastolic blood pressure)/3].

Exclusion criteria include; a history of cancers of the ovaries, endometrium, breast or those with benign conditions such as endometriosis, or multiple pregnancy. Those with medical disorders such as diabetes mellitus, chronic hypertension, and diseases of the liver, kidney, cardiovascular system or inflammatory conditions were also excluded. A structured questionnaire was used to gather age and clinical characteristics and a written informed consent was obtained from every participant. Ethical clearance was obtained from the Ministry of Health, North Kordofan State- Sudan, the Research Board at the Faculty of Medicine, the University of Khartoum and the University of Kordofan.

Five ml of venous blood was collected from both groups in plain tubes. Serum was obtained from centrifugation of the clotted samples and stored at -20°C until the assay. Cancer antigen-125 was measured for both patients and controls by an automated chemical analyzer (Mindary CL-1200i Chemiluminescence Immunoassay System-India) using antigen-antibody reaction. Serum CRP and UA levels were measured by (Mindary Bs200 Automated Benchtop Chemistry Analyzer-India) using enzymatic reaction according to the manufacturer’s instructions.

### Statistics analysis

Data was entered, coded and analyzed using a statistical package for social sciences version 20 software (SPSS Software, Chicago Inc., USA). Data was expressed as mean ± Standard Error of Mean (SEM). The t-test was used to compare the two groups (cases and controls). while One-way ANOVA was used to compare the variables of the three groups (control, mild, and severe preeclampsia). *P* value of <0.05 was considered significant. Pearson correlation was done to find correlation coefficient value (r) either positive (direct correlation) or negative (inverse correlation) with values < 0.3 representing no correlation, 0.3 - <0.5 representing weak correlations, 0.5 - < 0.7 represents moderate correlations and > 0.7 represents strong correlations. Multiple Receiver Operating Characteristic curve (ROC) was drawn to evaluate the validity of CA 125, CRP and UA in predicting pregnant women at risk of preeclampsia disease and its severity. The test was considered a good marker if it shows an area under the curve (AUC ≥0.8).

## Results

A total of 200 pregnant women: (100) cases of PE and (100) controls were recruited. The age and obstetric data of the studied women are presented in Table 1. Both groups were matched in age, body mass index (BMI) and gestational age among PE patients with the normotensive pregnant women. There was no significant difference (P>0.05) in the maternal age, BMI and gestational age. Mean levels of CA-125, CRP (P <0.001) and UA (P <0.05), were significantly higher in severe and mild PE in comparison with the controls as shown in Table 2. There was a strong positive correlation between CA-125, CRP and UA levels and mean arterial blood pressure (Table 3); (r> 0.7, P < 0.001).

**Table 1:** Age and clinical characteristics of the patient’s groups (mild and severe preeclampsia) and controls.

Variables	Controls	Mild PE	Severe PE	*P*-value
	(n = 100)	(n = 46)	(n = 54)	
	Mean ±SD	Mean ±SD	Mean ±SD	
Age(years)	26.67± 6.74	26.68± 6.31	26.33±7.47	0.987
BMI	26.78± 4.02	27.56± 4.97	27.93± 4.49	0.145
Gestational age (weeks)	33.67± 4.59	33.82± 3.81	34.46± 4.96	0.641

**Table 2:** Serum cancer antigen 125 (CA125), C-reactive protein (CRP) and uric acid (UA) and mean arterial blood pressure (MAP) in the patients and controls groups (mean± SEM).

Variables	Controls	Mild PE	Severe PE	*P*-value
	(n = 100)	(n = 46)	(n = 54)	
	Mean ±SEM	Mean ±SEM	Mean ±SEM	
CA-125	16.48± 0.584	21.94± 0.749	40.78± 1.336	< 0.001
CRP	4.79±0.178	15.17±0.788	31.50±1.709	< 0.001
UA	4.00±0.061	6.44±0.293	7.37±0.272	< 0.05
MAP	84.15±0.959	115.89±0.877	136.06±1.282	< 0.001

**Table 3:** Coefficient of correlations (r) of cancer antigen (CA-125), C-reactive protein (CRP) and uric acid (UA) with mean arterial blood pressure (MAP) in preeclampsia.

parameters	coefficient of correlations(r)	*P*-value
CA-125	0.771	< 0.001
CRP	0.808	
UA	0.708	

The study revealed a strong positive correlation between CA-125, CRP and UA levels and mean arterial blood; (r> 0.7, P < 0.001).

**Fig 1:**
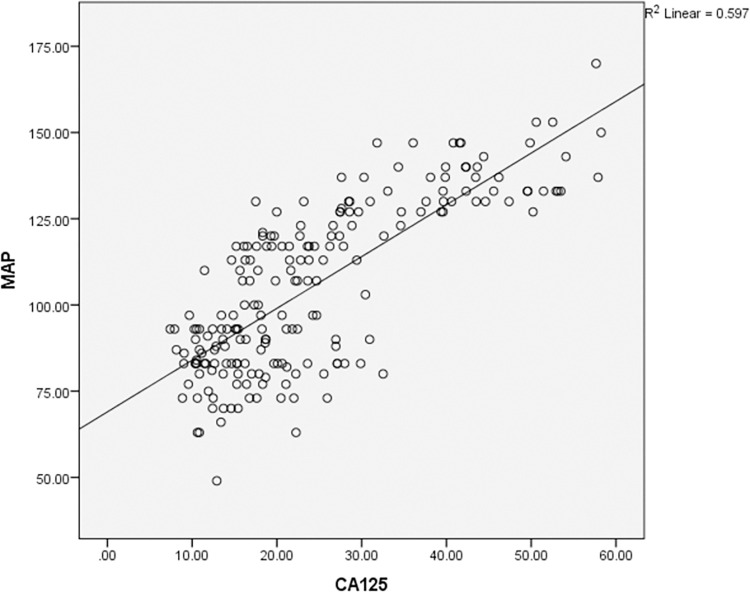
Correlation of cancer antigen 125 (CA-125) and mean arterial pressure (MAP) in the study groups (mild and severe preeclampsia). MAP = 40 + 2.333 CA-125 (regression equation according to coefficients table) (t = 17.11, p < 0.001).

**Fig 2:**
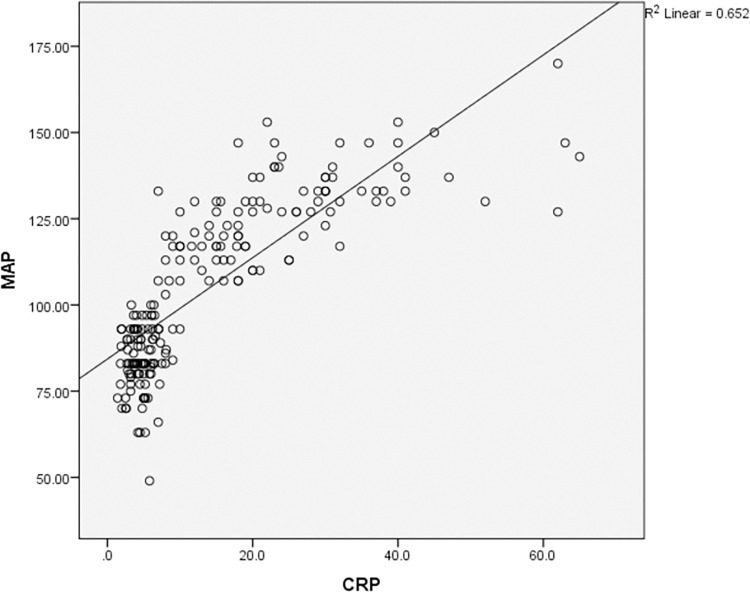
Correlation of C-reactive protein (CRP) and mean arterial pressure (MAP) in the study groups (mild and severe preeclampsia). MAP = 40 + 2 CRP (regression equation according to coefficients table) (t = 10.27, p < 0.001).

**Fig 3:**
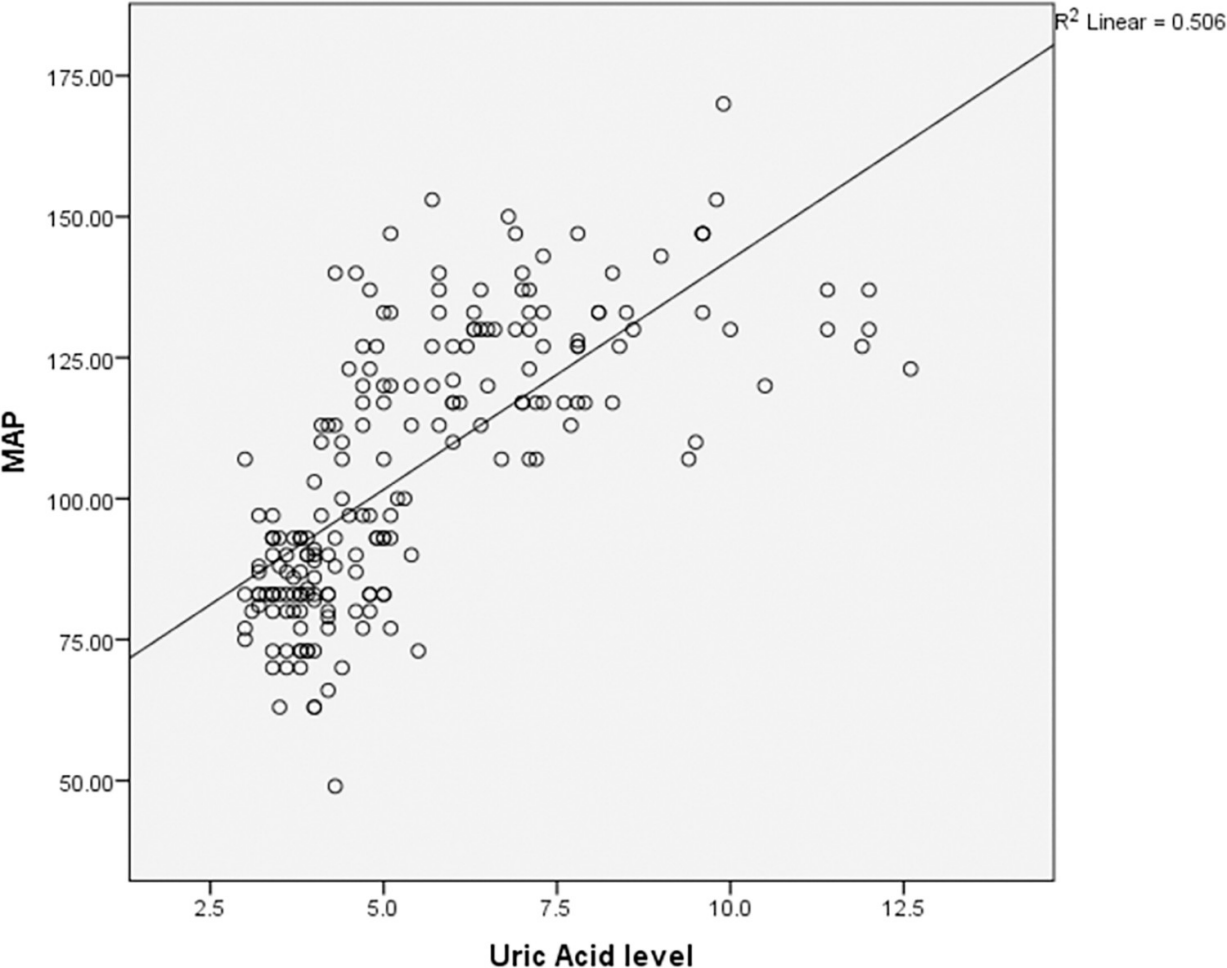
Correlation of uric acid (UA) and mean arterial pressure (MAP) in the study groups (mild and severe preeclampsia). MAP = 16.6667+11.6667 UA (regression equation according to coefficients table) (t = 14.24, p < 0.001).

**Fig 4:**
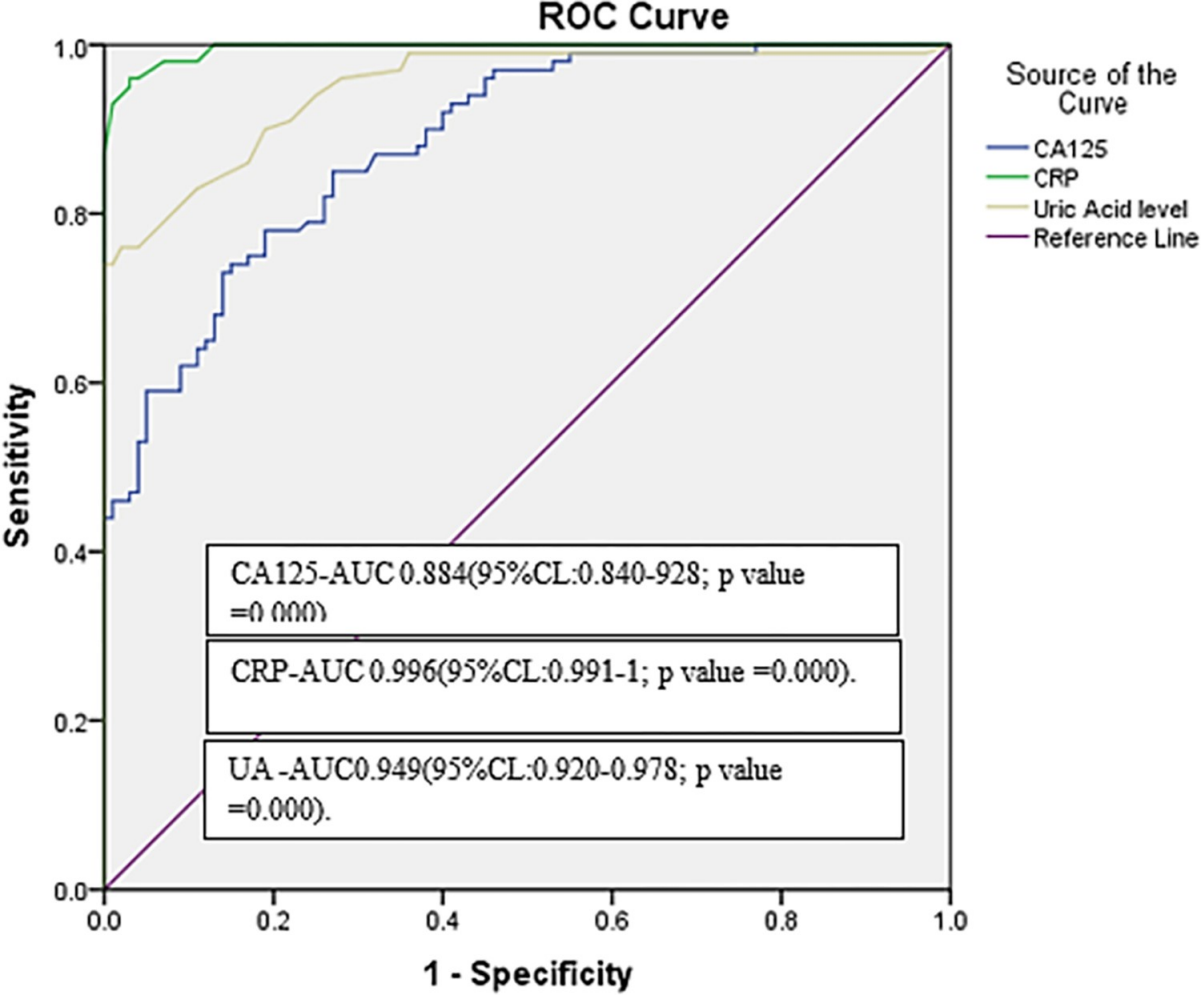
Receiver operating characteristic (ROC) curve of serum cancer antigen-125 (CA-125), C-reactive protein (CRP) and uric acid (UA) levels in preeclampsia.

According to the Pearson coefficient correlation test, MAP was found to have a significant effect on the increase in CA125, CRP and UA in preeclampsia as shown in Figs 1–3 respectively. Multiple Receiver Operating Characteristic (ROC) curves were drawn to evaluate the validity of CA-125, CRP and UA in predicting pregnant women at risk of preeclampsia disease and its severity as shown in Fig 4. The area under the curve for all three variables was more than 0.8. The sensitivity of CA-125 was (85%), the specificity was (73%), while the sensitivity of CRP was (95%), the specificity was (97%) and the sensitivity of UA was (86%), and the specificity was (83%).

## Discussion

In this study, the patient and control groups showed no significant differences concerning maternal age, gestational age and body mass index (BMI).

The study showed increased levels of CA-125, CRP inflammatory mediators, and UA among patients in comparison with the control group. This increase was directly correlated with the severity of PE. The significantly higher level of CA-125 in severe and mild PE in comparison with the control group (p<0.001) which was observed in the current study, coinciding with many previous studies [21, 23, 28] in which some authors suggested that CA-125 is a promising biochemical marker and can reflect the severity of preeclampsia [18, 21, 23, 28, 41]. This rise might be due to failure in trophoblastic invasion and the induction of an inflammatory process within the placenta that triggers the expression of CA-125 and is anticipated to be an essential mechanism for the elevation of serum CA-125 in preeclampsia. Contrary to these results Schröcksnadel et al. and Bon et.al found no statistically significant difference in CA-125 between patients and control groups [16, 20]. This discrepancy might be due to different sample sizes, demographic and genetic variations and different timing of CA-125 measurement during pregnancy. Furthermore, a strong positive correlation (r> 0.7, *P* < 0.001) between CA-125 levels and mean arterial blood pressure (MAP) in preeclampsia was reported (Fig 1). This finding is consistent with previous studies [28, 41].

Elevation in the mean levels of CRP in mild and severe PE patients, when compared with the control group, was also observed by other authors where Serum CRP was significantly positively correlated with MAP [24, 27–30]. As the systemic inflammatory response is one of the pathophysiological mechanisms of PE, CRP was more sensitive and specific to predicting PE in pregnant women. The increment in CRP was directly correlated with the severity of the disease and can be used for early prediction of the severity of PE. However, contradicting results [26] showed no association between the maternal inflammatory mediator CRP and established PE.

The significant difference in the mean values of UA in the patient’s group (mild and severe) in comparison with the control group showed a strong positive correlation with MAP. This elevated-serum uric acid level is consistent with other reported findings [34]. Soluble uric acid impairs the nitric oxide generation in endothelial cells inducing endothelial dysfunction and pre-eclampsia [37]. Recent evidence [34] supports a role for uric acid as a true cardiovascular risk factor, particularly for the development of hypertension and renal disease. The rise in uric acid levels in preeclampsia is due to placental injury, which causes purine catabolism and uric acid generation. However, this finding is contradicted by others who reported that a high level of serum uric acid was not consistently elevated in all women with severe preeclampsia, suggesting that the uric acid could not be a useful prognostic test as stated by Al Huri [39]. However, Kanti Mandal *et al*., Stated that serum UA and CRP may be possible to use as biomarkers for identifying women at risk of Preeclampsia [42].

According to the obtained findings of specificity and sensitivity of CRP and CA-125 levels in the current study, the potential role of these markers as a predictive test for severity of PE should be considered; since these markers are available and less expensive. Raised serum uric acid (UA) is one of the characteristic laboratory findings in preeclampsia. In clinical practice, serum UA measurement is considered to be a part of the workup in women with preeclampsia to monitor disease severity and assist in the management. The association between raised serum UA and preeclampsia has long been known [43]. Reduced UA clearance following reduction of glomerular filtration rate, increased reabsorption, and reduced secretion may be the origin of elevated serum levels in women with preeclampsia [33, 44]. Several studies have reported a positive correlation between elevated maternal serum UA and adverse maternal and fetal outcomes [32, 45]. The Receiver Operating Characteristic (ROC) curve of serum CA-125, CRP and UA in preeclampsia indicates the validity of these markers as sensitive and specific prognostic tools for the prediction of PE severity.

## Conclusions

There were increased levels of CA-125, CRP and UA levels in women with preeclampsia which were correlated with the severity of the disease. This study suggested that CRP and CA-125 are biochemical markers that reflect the severity of the inflammatory process in preeclampsia. Similarly, UA may be a useful biomarker for identifying women at risk of preeclampsia.
